# Impact of scanning errors on the trueness of digital implant impressions

**DOI:** 10.1186/s40729-026-00684-4

**Published:** 2026-04-28

**Authors:** Ragai-Edward Matta, Constantin Motel, Werner Adler, Manfred Wichmann, Felix Förtsch

**Affiliations:** 1 Department of Prosthodontics, Erlangen University Hospital, Glueckstrasse 11, 91054 Erlangen, Germany; 2 Department of Medical Informatics, Biometry and Epidemiology, Friedrich-Alexander-University of Erlangen-Nuremberg, Waldstrasse 6, 91054 Erlangen, Germany

**Keywords:** Implantology, Digital impression, Digital implant impression, Scanning errors

## Abstract

**Purpose:**

In dentistry, digital technologies have become firmly established in the field of oral implantology. The trueness of impressions taken using intraoral scanners as part of the digital workflow has a direct influence on the accuracy of fit of the subsequent restoration. Therefore, this in-vitro study investigates the influence of possible scanning errors on the trueness of digital implant impressions.

**Methods:**

A standardized titanium model of a maxillary alveolar ridge with three bone-level implants was digitized with two different scan body systems (Medentika and NT-Training) using an industrial high-precision scanner to generate the virtual reference models. Subsequently, three different modifications, including two different gingival heights and artificially created defects, were each digitized 15 times using two different intraoral scanners (Primescan and Trios 4) with both scan body systems. The digitized scan bodies were then matched with the scan body analogs in the Exocad software to generate the virtual working models. Trueness was examined by superimposing the virtual working models with the corresponding virtual reference model.

**Results:**

All scanning errors evaluated resulted in significantly higher deviations. Across all groups, the trueness decreased with increasing gingival height. The best results (30 ± 15 μm) were attained when scanning at the lower gingival height. The largest discrepancy (178 ± 63 μm) was found when impressions of the higher gingival height were taken.

**Conclusion:**

In the process of taking digital implant impressions, it is essential to maximize the exposure of the scan bodies. Furthermore, it is imperative to minimize defects in order to achieve optimal outcomes.

**Graphical abstract:**

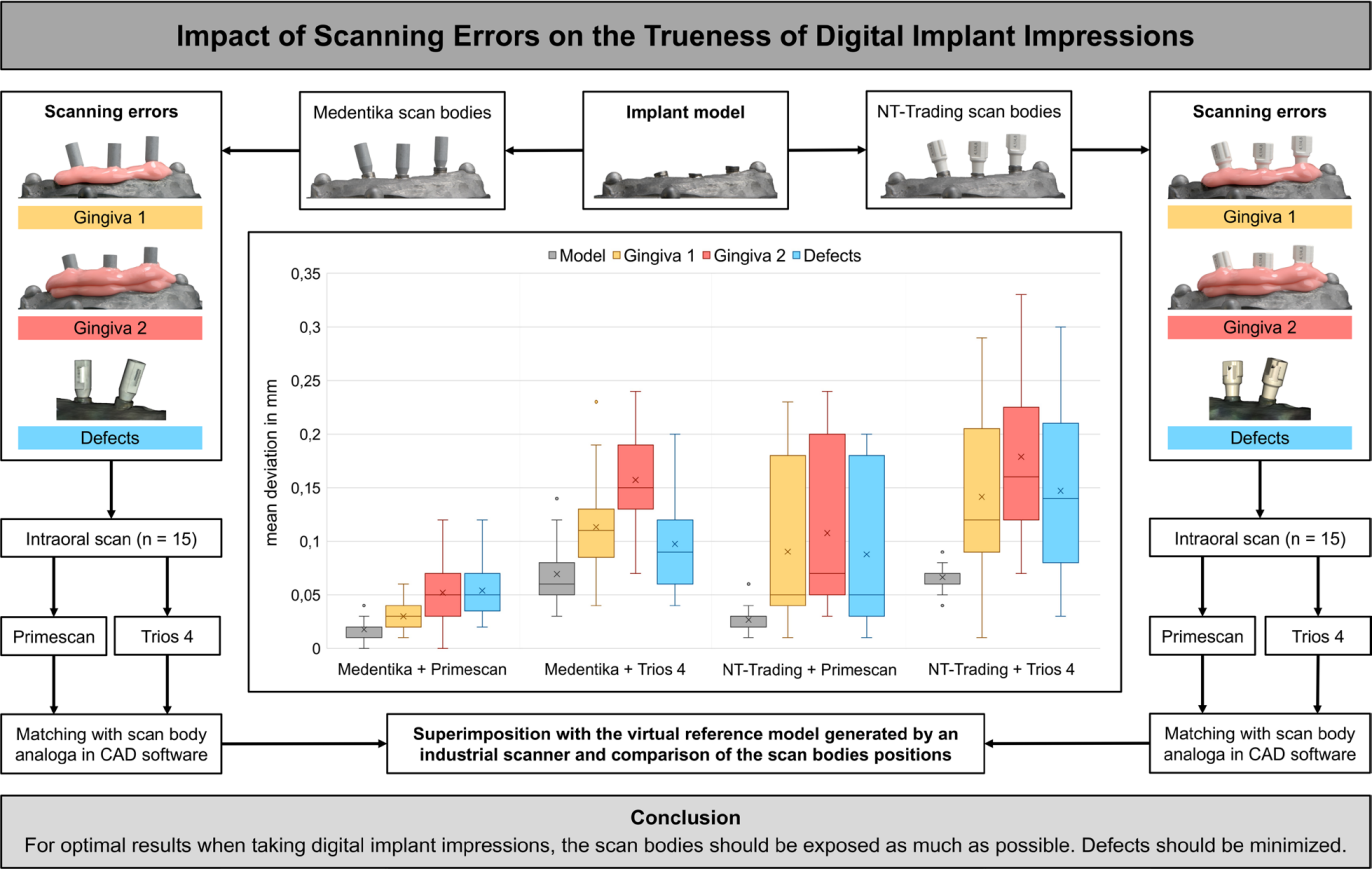

## Background

Dental implants have a wide range of indications, covering everything from the replacement of individual teeth and the restoration of edentulous jaw sections to the complete rehabilitation of edentulous patients. Both fixed and removable options are available, whereby the choice of restoration type must always be based on the individual patient situation [1–4]. Due to this wide range of indications and the excellent long-term prognosis of over 95% over a period of 10 years, regardless of the type of restoration chosen, dental implants are an integral part of the dental treatment spectrum [5].

The treatment process is divided into several steps, starting with the imaging to plan the number and position of the implants, followed by the surgical procedure. Subsequently, impressions are taken and the restoration is fabricated [6]. The impression-taking step plays a decisive role here, as the trueness of the impression has a direct influence on the precision of the model and thus on the precision of the subsequent prosthetic restoration [7]. Optimal accuracy of fit of the restoration is a fundamental prerequisite for maintaining the osseointegration of the implants, i.e. the structural and functional connection between the implant and the bone [8, 9]. This is the case because, unlike natural teeth, dental implants do not have a periodontium and are therefore unable to compensate for inaccuracies of the restoration, which can lead to both mechanical and biological complications [10, 11]. The biological complications range from pathological reactions of the soft and hard tissue as a result of increased plaque accumulation to implant loss due to peri-implantitis, while the mechanical complications include screw loosening, screw fractures or fractures of the implant or suprastructure [12, 13]. These possible consequences of inaccurate implant impressions illustrate the importance of this particular step in the implantology workflow.

Progressive digitalization is also making itself felt in the field of dental implantology through the use of CAD/CAM (computer-aided design/computer-aided manufacturing) technologies. Whereas treatment used to be based on conventional work steps such as two-dimensional imaging, traditional impression-taking of implants and the fabrication of restorations using the lost-mold technique [14, 15], it is now possible to carry out the entire treatment protocol using computer-assisted technologies. In this digital workflow, the impression is taken using an intraoral scanner, after which the dental restoration is designed in a CAD software [16, 17]. Current surveys show that more than half of the dentists in economically strong countries already use intraoral scanners, which points to the numerous advantages of this technology [18–20]. In addition to increased patient comfort, the benefits also include time and cost savings and the resulting overall increase in clinical efficiency [21, 22]. Regarding the trueness of the two procedures, the scientific data is currently inconsistent. While some studies find no significant difference [23–25], a large number of studies find that either conventional [26–28] or digital [22, 29, 30] implant impressions are more precise. In analogy to the impression posts in conventional impressions, so-called scan bodies are used to take the impression, which serve to transfer the implant position during the digital impression. During the subsequent design of the dental restoration, the scan bodies are superimposed with the model analogs from the digital implant library of the CAD software to create the digital working model [31]. Today, implant restorations are predominantly fabricated utilizing a CAD software, even when conventional impressions are taken. In order to accomplish this objective, a plaster model with implant analogs is produced based on the impression. Subsequently, the model undergoes digitization through the use of a laboratory scanner utilizing specialized scan bodies. As with the fully digital workflow, the digitized scan bodies must be matched with the corresponding model analogs in the CAD software to generate the virtual working model, which is then used to design the restoration [32–34]. This crucial step in the implant treatment procedure is a significant source of error, yet it is frequently omitted from studies examining the accuracy of implant impressions. Consequently, this oversight may lead to an underestimation of deviations [24, 35–39].

Although the feasibility and trueness of digital implant impressions have already been extensively studied, there is a lack of data on the influence of scanning errors [40]. A few existing studies on this topic found no influence of the exposure length of the scanbodies on the trueness of the digital implant impression, whereby the unprocessed intraoral scan was always used for comparison in these studies [37, 39, 41]. This prompts further inquiry into the impact of scan body exposure length and scanning errors on the superimposition of scan bodies with their corresponding analogs within the CAD software, and the subsequent effect on the trueness. Therefore, the null hypothesis of this study is that scanning errors in digital implant impressions have no influence on the trueness of the recording of the implant position.

## Materials and methods

**Fig. 1:**
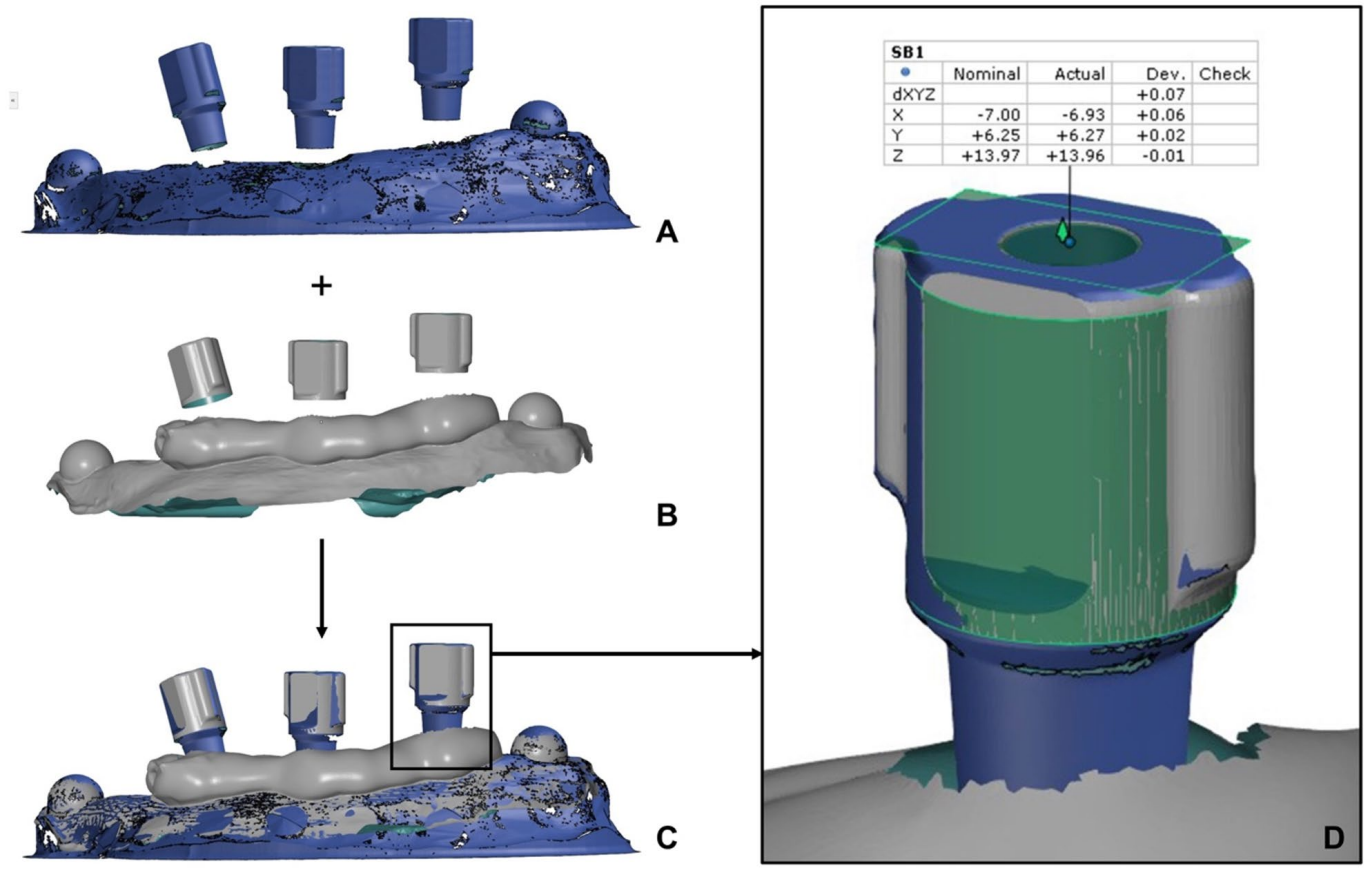
Matching of the virtual reference model with the Exocad STL file using the example of the Gingiva 1 modification and impression taking with the NT-Trading scan bodies **A** Virtual reference model with NT-Trading scan bodies **B** Virtual working model (Exocad STL file) of the Gingiva 1 modification with NT-Trading model analogs **C** Superimposition of the two virtual models **D** Comparison of the scan body position points using the example of the most anterior scan body

**Fig. 2:**
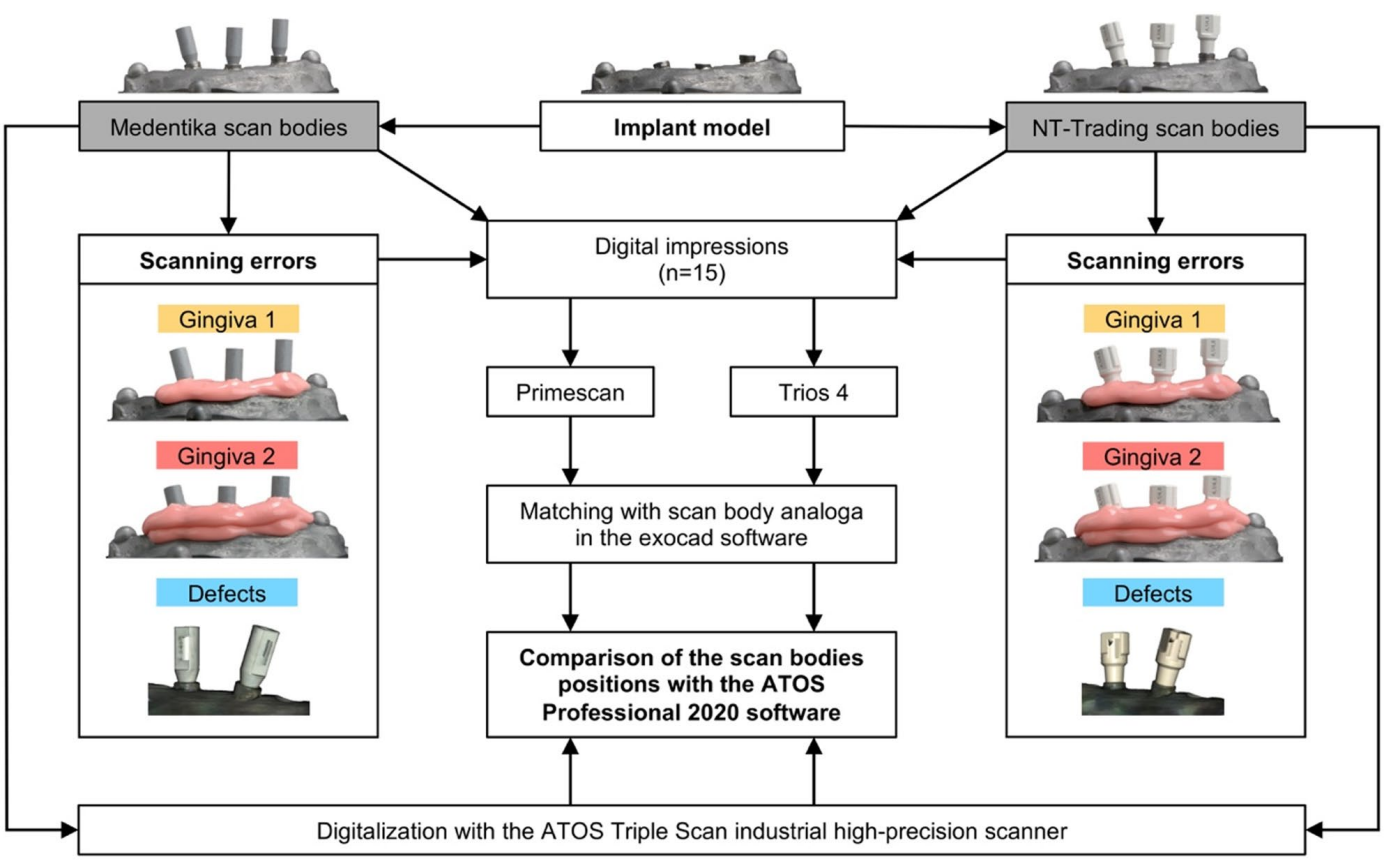
Overview of the study design

The implant model used for this study was a titanium model of a standardized maxillary alveolar ridge with three bone-level implants, with the most distal implant inserted at an angulation of 15°. Two different scan body systems were selected for the digital impressions: Medentika L-Series L1410 (Straumann GmbH, Freiburg im Breisgau, Germany) and NT-Trading L-Series L 9.S3D4.148 (NT-Trading GmbH & Co. KG, Karlsruhe, Germany). The reference model was digitized with both scan body systems using an industrial high-precision scanner (ATOS Triple Scan, Carl Zeiss GOM Metrology GmbH, Braunschweig, Germany), which has an average measurement error of only 3 μm [42]. The virtual reference models were saved as STL files (standard tessellation language) for later comparison of the scan bodies positions. The digital impressions were then performed using the two intraoral scanners CEREC Primescan (Dentsply Sirona, Charlotte, USA) and Trios 4 (3Shape, Copenhagen, Denmark). First, the reference model was scanned 15 times for both scan body systems. The scanning errors were then simulated, including two different gingival heights and artificially generated defects. For the simulation of the two different gingival heights, the material Gingifast Rigid (Zhermack SpA, Rovigo, Italy) was built up in two steps of approximately 3 mm each. For gingival build-up, 3 mm were measured on each scan body and marked to define the silicone build-up height. The silicone was then applied from the mixing cannula in a strand with a diameter of 5 mm. For the defects, a defect was created palatally on each of the two distal scanbodies by cutting out a geometrically defined shape. All modifications were also scanned 15 times with both intraoral scanners utilizing both scan body systems. All scans were performed by the same experienced clinician. The scan path was selected in accordance with the manufacturers’ recommendations, and the intraoral scanners were recalibrated before scanning each modification for every combination of intraoral scanner and scan body system. All intraoral scans were exported as STL files for subsequent processing in the CAD software. In the Exocad software (Tizian Creativ RT DentalCAD 3.2 Elefsina by Exocad, Schuetz Dental GmbH, Rosbach, Germany), the scan bodies were superimposed with the corresponding model analogs from the digital implant library according to the best-fit principle. The virtual working model created in this way was exported again as an STL file. The superimposition of the virtual reference and working models and the subsequent comparison of the scan bodies positions was carried out in the GOM Inspect Professional 2020 software (Carl Zeiss GOM Metrology GmbH). In the first step, a uniform coordinate system was defined, with the x-axis describing the deviation in the mesiodistal direction, the y-axis the deviation in the orovestibular direction and the z-axis the deviation in the craniocaudal direction. The comparison of the scan body positions was performed by creating identical and reconstructable points in the center of each cranial scan body surface. These points were created by generating best-fit cylinders and planes. Subsequent to these preparatory procedures, the two virtual models were initially subjected to automatic superimposition. The precise superimposition was executed utilizing a best-fit superimposition approach, wherein the alveolar ridge was selected as the superimposition surface, with the scan bodies deliberately excluded. This method was chosen because the deviations of the implant positions could be falsely underestimated if the scan body surface was included for superimposition. The three-dimensional deviation (xyz axis) and the deviations in all three axes of the coordinate system were determined by comparing the scan body position points of the virtual working model and the reference model, whereby the mean value was always calculated from the deviations of the three individual scan bodies (Fig. 1). The statistical analysis was carried out using the R software (R Core Team, R Foundation for Statistical Computing, Vienna, Austria). After assessment of normality using the Shapiro–Wilk test, a Wilcoxon rank-sum test was performed. *P* values were adjusted using the Holm correction within each combination of intraoral scanner and scan body system. The significance level was set at 0.05. Figure 2 shows an overview of the study design.

## Results

A detailed examination of the three-dimensional deviations (xyz axis) revealed that all scanning errors investigated resulted in higher deviations, irrespective of the scan body system or intraoral scanner utilized (Table 1).

**Table 1:** Descriptive statistics of the deviation in the xyz axis for all combinations of scan body system and intraoral scanner: Mean deviation (Mean) with standard deviation (SD) and the minimum (Min) and maximum (Max) deviation in µm

Group	Mean ± SD [Min–Max] (µm)			
	Model	Gingiva 1	Gingiva 2	Defects
Medentika + Primescan	18 ± 8 [0–40]	30 ± 15 [10–60]	52 ± 26 [0–120]	54 ± 24 [20–120]
Medentika + Trios 4	70 ± 26 [30–140]	113 ± 39 [40–230]	157 ± 39 [70–240]	98 ± 45 [40–200]
NT-Trading + Primescan	27 ± 12 [10–60]	90 ± 72 [10–230]	108 ± 75 [30–240]	88 ± 73 [10–200]
NT-Trading + Trios 4	66 ± 10 [40–90]	142 ± 71 [10–290]	179 ± 63 [70–330]	147 ± 70 [30–300]

**Fig. 3:**
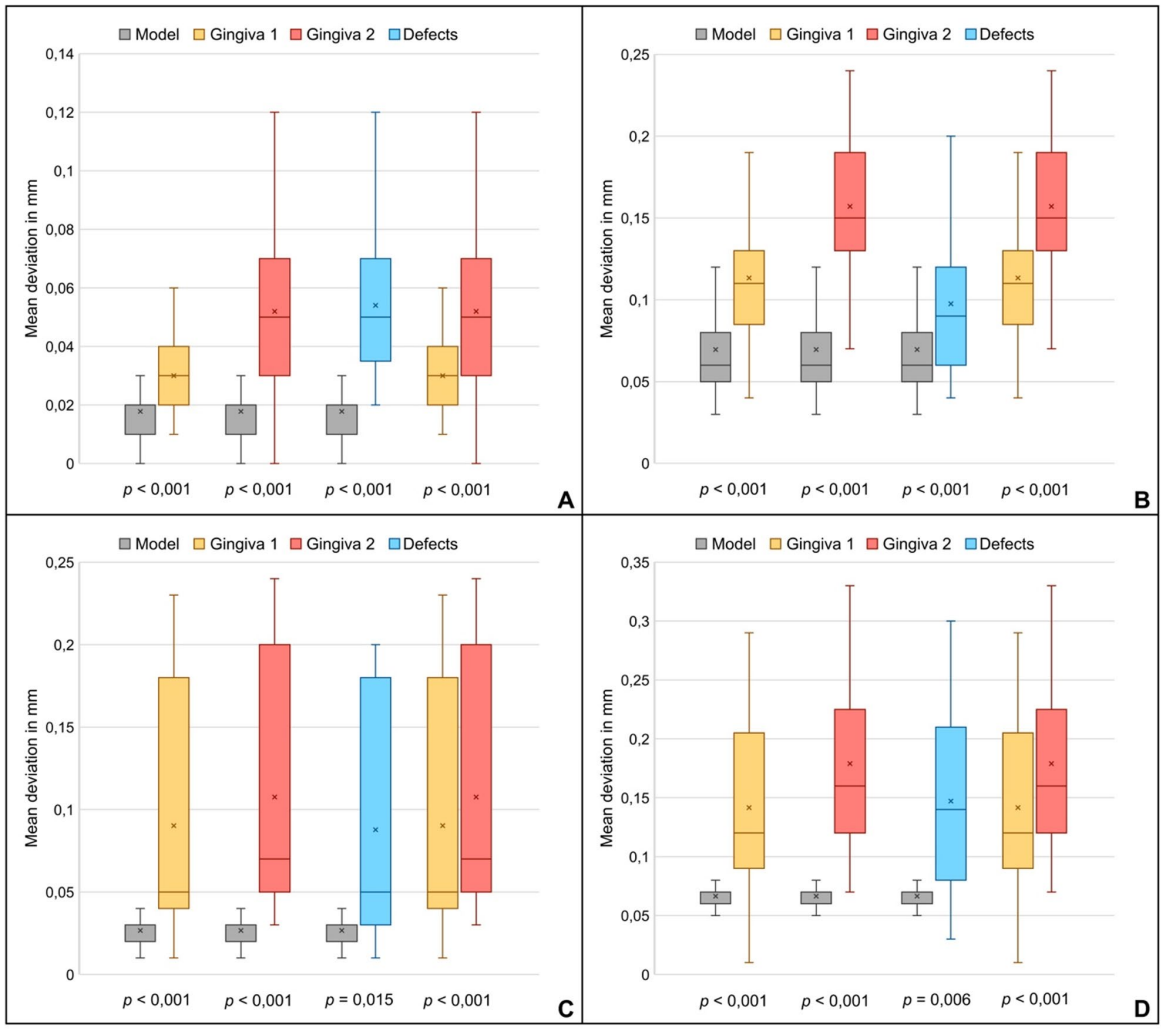
Box-whisker plots showing the mean deviations in mm to visualize the comparison of all investigated scanning errors in the xyz-axis **A** Medentika scan bodies and Primescan **B** Medentika scan bodies and Trios 4 **C** NT-Trading scan bodies and Primescan **D** NT-Trading scan bodies and Trios 4

The statistical comparisons of all scanning errors with the reference model, as well as the comparisons of the two simulated gingival heights, were always statistically significant (Fig. 3). The impressions captured using the CEREC Primescan in conjunction with Medentika scan bodies demonstrated the highest trueness across all modifications, with the modification Gingiva 1 achieving the most precise results, showing a deviation of 30 ± 15 μm. The least accurate results were obtained for the impression of the modification Gingiva 2 with the combination of NT-Trading scan bodies and Trios 4, exhibiting a deviation of 179 ± 63 μm. In this combination, the defects also had the greatest influence with a deviation of 147 ± 70 μm. The difference between the two simulated gingival heights was the greatest for impressions made with the NT-Trading scan bodies and the Trios 4 scanner with a difference in the mean deviations of 44 μm. In a comparison of the two scan body systems, the scans with the Medentika scan bodies were always more accurate, with the only exception being the impressions of the initial model taken with the Trios 4 scanner. When comparing the two intraoral scanners used, the Primescan scanner proved to be more precise across all modifications regardless of the scan body system used. When looking at the individual axes, the same results were obtained as for the three-dimensional deviation; the descriptive statistics for the individual axes can be found in Tables 2, 3 and 4.

**Table 2:** Descriptive statistics of the deviation in the x axis for all combinations of scan body system and intraoral scanner: Mean deviation (Mean) with standard deviation (SD) and the minimum (Min) and maximum (Max) deviation in µm

Group	Mean ± SD [Min–Max] (µm)			
	Model	Gingiva 1	Gingiva 2	Defects
Medentika + Primescan	10 ± 9 [0–40]	14 ± 13 [0–60]	30 ± 20 [0–80]	30 ± 22 [0–90]
Medentika + Trios 4	36 ± 35 [0-140]	64 ± 49 [10–210]	98 ± 52 [0–190]	70 ± 53 [0–190]
NT-Trading + Primescan	12 ± 12 [0–60]	80 ± 73 [0–220]	95 ± 80 [20–230]	70 ± 70 [0–190]
NT-Trading + Trios 4	18 ± 15 [0–60]	112 ± 76 [0–280]	122 ± 48 [10–220]	126 ± 71 [20–280]

**Table 3:** Descriptive statistics of the deviation in the y axis for all combinations of scan body system and intraoral scanner: Mean deviation (Mean) with standard deviation (SD) and the minimum (Min) and maximum (Max) deviation in µm

Group	Mean ± SD [Min–Max] (µm)			
	Model	Gingiva 1	Gingiva 2	Defects
Medentika + Primescan	6 ± 7 [0–20]	20 ± 16 [0–60]	33 ± 23 [0–90]	23 ± 18 [0–80]
Medentika + Trios 4	25 ± 18 [0–70]	34 ± 26 [0–110]	52 ± 32 [0–120]	36 ± 20 [0–90]
NT-Trading + Primescan	17 ± 15 [0–60]	32 ± 20 [0–80]	27 ± 22 [0–100]	46 ± 31 [0–100]
NT-Trading + Trios 4	21 ± 12 [0–50]	42 ± 29 [0-110]	55 ± 36 [0–160]	44 ± 28 [0–100]

**Table 4:** Descriptive statistics of the deviation in the z axis for all combinations of scan body system and intraoral scanner: Mean deviation (Mean) with standard deviation (SD) and the minimum (Min) and maximum (Max) deviation in µm

Group	Mean ± SD [Min–Max] (µm)			
	Model	Gingiva 1	Gingiva 2	Defects
Medentika + Primescan	8 ± 8 [0–40]	9 ± 9 [0–40]	16 ± 12 [0–40]	33 ± 17 [0–100]
Medentika + Trios 4	42 ± 19 [0–80]	72 ± 30 [10–150]	96 ± 33 [10–160]	43 ± 18 [0–80]
NT-Trading + Primescan	7 ± 7 [0–20]	12 ± 10 [0–40]	18 ± 11 [0–50]	10 ± 8 [0–30]
NT-Trading + Trios 4	56 ± 13 [20–80]	61 ± 26 [10–120]	101 ± 65 [10–230]	52 ± 16 [20–90]

## Discussion

The superimposition of intraoral scans with a virtual reference model in an analysis software to analyze the trueness of digital implant impressions has been published extensively and is therefore an established scientific evaluation method [24, 35, 36, 40, 43]. With its low average measurement error of just 3 μm [42], the ATOS II Triple Scan high-precision scanner used in this study remains below the values of high-precision scanners used in comparative studies [24, 36] and offers a broad range of measurement options in the connected GOM Inspect Professional analysis software. This evaluation method has been published on numerous occasions due to its notable applicability and substantial variability [44–47].

The null hypothesis of the present study was that scanning errors have no influence on the trueness of digital implant impressions. The null hypothesis must be rejected, as all modifications examined led to significantly higher deviations (Table 2).

The trueness of digital implant impressions is known to decrease with a shorter exposure length of the scan bodies, as evidenced by comparative studies [38, 48]. In the comparative study by Giménez-González et al., deviations between 5 ± 13 μm and 27 ± 51 μm were found. Some of these deviations are significantly lower than the values obtained from this study, which ranged from 18 to 179 μm (Table 1). These smaller discrepancies can potentially be attributed to variations in the evaluation methodology, as the study by Giménez-González et al. did not involve the comparison of implant positions through digitizing and superimposing the reference model. In this study, the position was compared by measuring the distance with a coordinate measuring machine. In this process, a scan body was defined as the origin of the coordinate system [38]. Consequently, the deviation of this scan body is not considered, potentially resulting in lower deviation values. Additionally, the unprocessed intraoral scans were utilized for comparative analysis. Consequently, the supplementary deviation induced by the matching process in the CAD software [33] was not considered in the analysis. Another reason could be the difference in the initial models, since in the study by Gimenez-Gonzalez et al. a model with six scan bodies was digitized, of which four scan bodies were always maximally exposed. As a result, there were always many areas for superimposing the individual images during the intraoral scan [38]. In a similar comparative study by Gomez-Polo et al., which employed the same evaluation methodology as Gimenez-Gonzalez et al., discrepancies were identified between 69 ± 39 μm and 267 ± 259 μm. In this study, a model with six implants was also digitized using an intraoral scanner. However, in this case, the scan bodies were not maximally exposed in any of the study groups, which could be responsible for the higher deviations [48]. In addition to these two comparative studies, some investigations have identified that the exposure length of the scan bodies does not influence the trueness of digital implant impressions [37, 39, 41]. Furthermore, no studies have identified superior trueness with a shorter exposure length. To contextualize these findings, it is crucial to acknowledge that none of the comparative studies incorporated matching in CAD software. Consequently, the supplementary error resulting from this process [33] was not considered in the analysis.

In addition to the gingival height, this study also investigated the influence of defects in the process of capturing the scan bodies. The artificially generated defects resulted in significantly higher deviations across all study groups (Table 1; Fig. 3). The issue of defects in digital implant impressions has been the subject of only a limited amount of research to date, as evidenced by the paucity of comparable studies available in the literature [40]. This study by Park et al. confirms that defects when capturing scan bodies lead to higher deviations; however, the exact results are difficult to compare due to several study characteristics. In the comparative study, only a single implant with a scan body and no associated implant model was digitized. Furthermore, different sizes and geometries of the defect were simulated as an exact percentage of the scan body’s surface area [40].

An acceptable misfit for implant prosthodontics is frequently reported in the literature as 150 μm [49, 50]. This threshold was exceeded only for the Gingiva 2 modification when using the TRIOS 4 scanner in combination with NT-Trading scan bodies; all other study groups remained below this value. However, direct clinical transferability is limited, because the present study did not evaluate the entire workflow but rather focused solely on the digital impression and the subsequent superimposition of the scan bodies with the corresponding model analogs in the CAD software. Nevertheless, the findings allow valuable conclusions, as errors should be minimized at each procedural step to keep the likelihood of complications as low as possible.

In addition to comparing the scanning errors, the study data also allows a comparison of the two intraoral scanners and scan body systems used. The lower deviation when using the Primescan scanner is clearly evidenced by a direct comparison of the two intraoral scanners used. A detailed examination of the three-dimensional deviation (xyz axis) revealed that the Primescan scanner exhibited lower deviations for all modifications examined, irrespective of the scan body system employed. This observation is corroborated when analyzing the results in relation to the individual axes of the coordinate system. The sole exception was the impression of the defects modification using the NT-Trading scan bodies, in which the Trios 4 scanner exhibited a 2 μm lower mean deviation in the y-axis. The present findings are consistent with the current literature on the subject, as the Primescan scanner has consistently demonstrated lower deviations than the Trios 4 scanner in comparative studies on the accuracy of different intraoral scanners [51–53].

When comparing the two scan body systems in regard to the three-dimensional deviation, the scans using Medentika scan bodies proved to be more accurate in seven of the eight possible combinations of modification and intraoral scanner. The Medentika scan bodies also performed better in seven comparisons on the x and y axes, and in six of the eight comparisons on the z axis. This result confirms the findings of a recent systematic review by Mohajerani et al., which found cylindrical scan body shapes to be advantageous in terms of scanning accuracy [54].

Despite the promising results of this study, its limitations must also be taken into account. It is important to acknowledge that this study was conducted in a laboratory setting. This methodological choice precluded the consideration of patient-specific challenges associated with digital impression taking, such as saliva, lighting, or mouth opening. Furthermore, the gingiva was built up freehand to simulate different gingival heights. Although a guideline value of 3 mm was adhered to, this limits the comparability with other studies. A similar limitation arises with the defects, as these were created according to a geometrically defined shape, but the exact defect size in relation to the entire scan body surface was not determined. This also limits the comparability with other studies. The only software used to match the scan bodies with the corresponding model analogs in the CAD software was exocad, as this is an open software and therefore allowed the STL file to be re-exported. However, given the extensive range of CAD software programs currently available, restricting the study to a single program may limit its overall relevance and applicability.

## Conclusion

Taking into account the limitations of the presented study, the following conclusions can be drawn:

- For the best results in digital implant impressions, it is imperative that the scan bodies be exposed to the greatest extent possible.
- To achieve the highest possible trueness, efforts should be made to minimize the occurrence of defects during the scanning process.
- A cylindrical scan body shape has a positive effect on trueness in digital implant impression.

## Data Availability

The underlying data is available from the corresponding author upon reasonable request.

## Abbreviations

CAD: Computer aided design
CAM: Computer aided manufacturing
STL: Standard tessellation language
